# Establishment of a prediction model for the impact of endometrial thickness on the day of embryo transfer on ectopic pregnancy in frozen-thawed embryo transfer cycle

**DOI:** 10.3389/fendo.2023.1259608

**Published:** 2023-11-06

**Authors:** Qiuyuan Li, Xiyuan Deng, Ahui Liu, Haofei Shen, Xuehong Zhang

**Affiliations:** ^1^ The First School of Clinical Medicine, Lanzhou University, Lanzhou, Gansu, China; ^2^ Reproductive Medicine Center, The First Hospital of Lanzhou University, Lanzhou, Gansu, China; ^3^ Gansu Province Prenatal Diagnosis Center, Key Laboratory of Prevention and Control of Birth Defects of Gansu Province, Gansu Provincial Maternity and Child-Care Hospital/Central Hospital of Gansu Province, Lanzhou, China; ^4^ Department of Obstetrics and Gynecology, the First Hospital of Lanzhou University, Key Laboratory of Gynecologic Oncology of Gansu Province, Lanzhou, China; ^5^ Key Laboratory for Reproductive Medicine and Embryo, Lanzhou, Gansu, China

**Keywords:** endometrial thickness, frozen-thawed embryo transfer, ectopic pregnancy, nomogram prediction model, ROC

## Abstract

**Objective:**

This study aims to investigate the factors affecting the ectopic pregnancy (EP) rate in the frozen-thawed embryo transfer (FET) cycle.

**Methods:**

This study retrospectively analyzed 5606 FET cycles, including 5496 cycles resulting in intrauterine pregnancy and 110 cycles resulting in EP. Smooth curve fitting and piece-wise linear regression were utilized to evaluate a non-linear association between endometrial thickness (EMT) and EP. Multiple logistic regression analysis was used to study the effect of EMT on the embryo transfer (ET) day and other indexes on EP rate after adjusting for confounding factors. A nomographic prediction model was employed to predict EP occurrence. The predictive efficacy of the model was assessed using the area under the receiver operating characteristic (ROC) curve (AUC), utilizing the bootstrap sampling method for internal validation.

**Results:**

After accounting for the confounding factors, the segmented linear regression analysis indicated that the EMT inflection point was 9 mm; the EP rate significantly decreased by 28% with each additional millimeter of EMT up to 9 mm (odds ratio (OR) = 0.72; 95% confidence interval (CI), 0.53–0.99; *P* = 0.0412) while insignificantly decreased when the EMT was greater than 9 mm (OR = 0.91; 95% CI, 0.76–1.08; *P* = 0.2487). Multivariate logistic regression analysis revealed that after adjusting for confounders, EP risk significantly increased in the number of previous EPs ≥ 1 (OR = 2.29; 95% CI, 1.26–4.16; *P* = 0.0064) and tubal factor infertility (OR = 3.86; 95% CI, 2.06–7.24; *P* < 0.0001). Conversely, EP risk was significantly reduced by the increased EMT (OR = 0.84; 95% CI, 0.74–0.96; *P* = 0.0078) and the blastocyst transfer (OR = 0.45; 95% CI, 0.27–0.76; *P* = 0.0027). These variables were used as independent variables in a nomogram prediction model, resulting in an AUC of 0.685. The nomination models were internally verified using self-sampling (bootstrap sampling resampling times = 500). This validation yielded an AUC of 0.689, with a sensitivity of 0.6915 and a specificity of 0.5790. The internal validation indicated minimal fluctuations in the AUC, signifying a relatively stable model.

**Conclusion:**

Undergoing EMT on the day of ET poses a separate EP risk in the FET cycle; to mitigate the EP incidence, the EMT should exceed 9 mm before ET. Furthermore, previous EPs and tubal factor infertility were additional factors independently increasing EP risk. Furthermore, implementing blastocyst transfer demonstrated that EP incidence was significantly reduced. Utilizing a nomogram predicting system enables EP risk evaluation before ET for individual patients, establishing a basis for devising clinical strategies for ET.

## Introduction

Ectopic pregnancy (EP) is a complication of assisted reproductive technology (ART), where the embryo implants outside the uterus. EP incidence rate following ART (2%–5%) (1) is significantly greater than that of spontaneous conception (1%–2%) (2–4), resulting in unsuccessful pregnancies and jeopardizing the patients’ lives and emotional and financial stress. Although the increased EP incidence following ART could be related to an excessively high hormone environment (5) or factors related to the fallopian tube (6, 7), embryo (3, 8), or *in vitro* fertilization (IVF) (9), the precise reasons remain unclear and require further clinical investigation to minimize this incidence.

Measuring endometrial thickness (EMT) using transvaginal ultrasound is utilized to assess endometrial receptivity and ascertain the embryo transfer (ET) timing due to its non-intrusive, straightforward, cost-effective, and repeatable benefits (10–12). However, earlier research on the impact of EMT on EP rate has conflicting outcomes. Several retrospective studies revealed that a thinner endometrium is a separate EP risk factor (13–16). Additional meta-analyses indicated an insignificant association between EMT and EP (17, 18). Consequently, further research is required to investigate the impact of EMT on EP during the frozen-thawed embryo transfer (FET) cycle.

This research established the cut-off point at which EMT impacts EP in FET by retrospectively analyzing our center’s data. We also assessed EP incidence for each patient by constructing a predictive model to support clinical ET protocol development.

## Materials and methods

### Study design and participants

**Figure 1:**
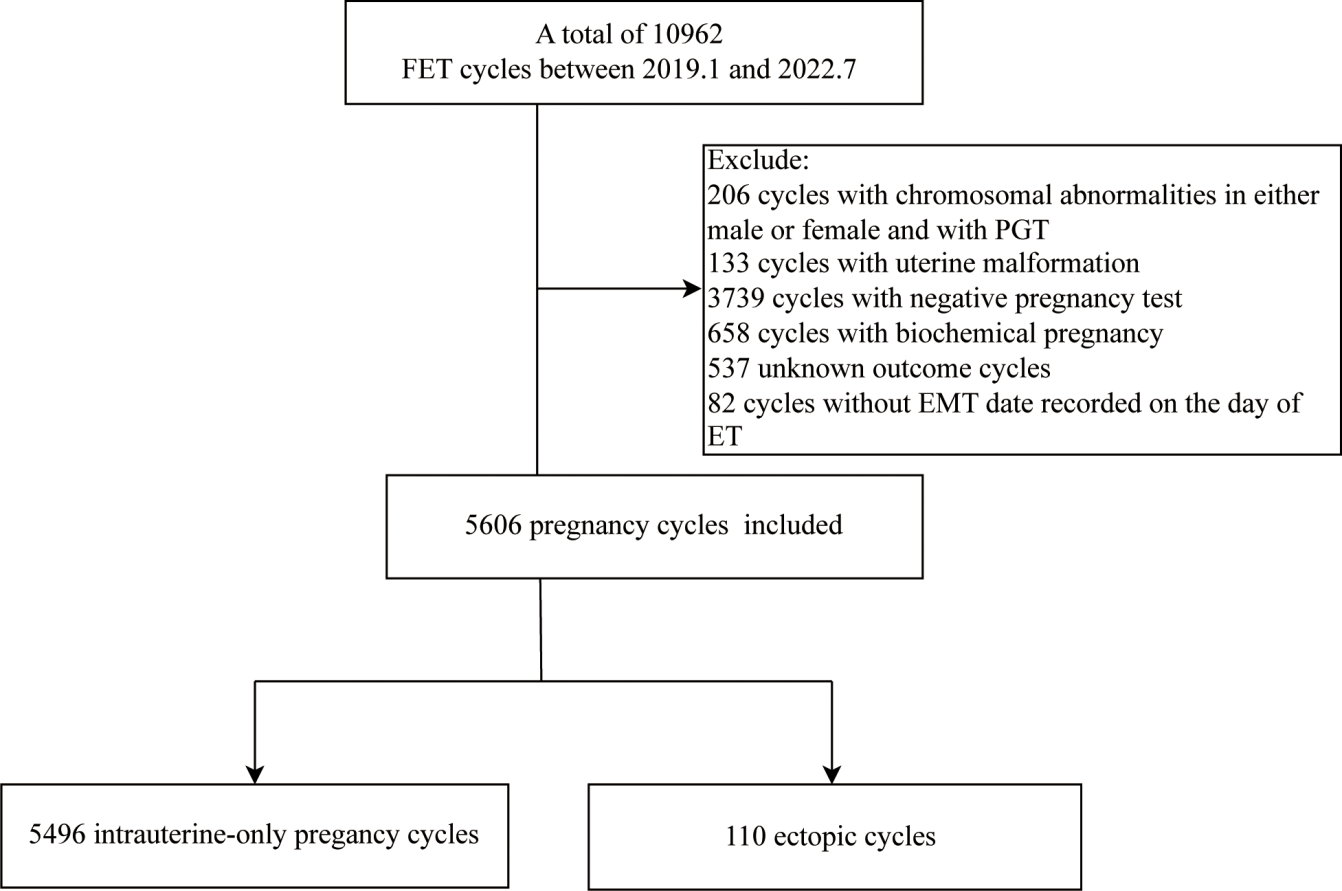
Study flow chart.

A total of 5606 FET cycles, 5496 cycles resulting in intrauterine pregnancy and 110 cycles resulting in EP, were analyzed at the Reproductive Center of the First Hospital of Lanzhou University from January 2019 to July 2022, with an overall EP rate of 1.96%. The Ethics Committee of the First Hospital of Lanzhou University approved this study (Ethical approval number: LDYYSZLLKH2023-07), with no requirement for signing the informed consent. The exclusion criteria included males or females with chromosomal abnormalities and pre-implantation genetic testing (PGT) cycles, uterine malformation, negative pregnancy test, biochemical pregnancy, and key data deletion cycle, representing the study flowchart in **Figure 1**.

### Endometrial preparation and embryo evaluation

In the Gonadotropin-releasing hormone-agonist hormone replacement therapy (GnRHa+HRT) cycle, patients with irregular menstrual cycles, ovulation disorders, or unsuccessful prior preparation programs were intramuscularly injected with 1.875 mg of long-acting triptorelin acetate (Ipsen Pharma Biotech, France) on the second–third day of the menstrual cycle. Twenty-eight days after the triptorelin injection, 4–6 mg/daily of estradiol valerate (Progynova, Bayer Group, Germany) was administered orally. Subsequently, the estradiol valerate dose was modified based on EMT and specific conditions of follicular development. After 12–14 days, when EMT was ≥ 8.0 mm, E2 was > 100 pg/mL, P was < 1 ng/mL, luteal support was administrated to transform endometrium: 30 mg/daily of oral dydrogesterone (Duphaston, Solvay Pharmaceuticals Ltd, Netherlands) plus 600 mg/daily of vaginal progesterone soft capsules (Urtogestan, Besins-healthcare, French); when E2 level was low, the estradiol dosage was adjusted; when P was ≥ 1 ng/mL, the transplantation was canceled. Cleavage-stage embryos were transferred on the third–fourth day of transforming endometrium, and blastocysts were transferred on the sixth–seventh day. Luteal support continued until the human chorionic gonadotropin (HCG) detection (serum β-hCG ≥ 50 U/L indicated positive HCG on the fourteenth day following ET); when the pregnancy was successful, the luteal support lasted until 12 weeks of pregnancy. When estradiol valerate was administrated until HCG detection, the dosage was gradually decreased until withdrawal.

In the HRT cycle, individuals experiencing irregular menstruation, ovulation disorder, or a thin endometrium were orally administrated 4–6 mg of estradiol valerate daily, starting on the second–third day of their menstrual cycle. Following a 12–14 day treatment, when the EMT was ≥ 8.0 mm, the luteal support, and ET timing remained consistent with the GnRHa+HRT cycle.

In the natural cycle (NC) cycle, patients with normal menstruation and no ovulation disturbance were examined by ultrasound every two to three days from the eighth to the tenth day of the menstrual cycle to monitor EMT and follicle size. Combining the E2, P, and LH levels determined the ovulation date when the follicle diameter reached 17 mm or more and the EMT was 8.0 mm or higher; if necessary, HCG was administered to induce ovulation. After ovulation, luteal support was given to induce intima transformation into the secretory phase: Cleavage-stage embryos were transferred on the second–third day of the transformed endometrium, and blastocysts were implanted into the uterine cavity on the fifth–sixth day after transformation. Luteal support remained consistent with the GnRHa+HRT cycle.

In the ovulation induction cycle, the ovaries were subjected to mild stimulation using low-dose oral ovulation-inducing medications (letrozole) or gonadotropin (Gn). The follicular development and EMT were monitored by transvaginal ultrasound. HCG was administered when the follicle size was equal to or greater than 17 mm, and the EMT was equal to or greater than 8.0 mm by considering E2, P, and LH levels. After ovulation, luteal support was administered to induce intima transformation to the secretory phase. Luteal support and ET timing were like those in the NC cycle.

The transferred embryos were evaluated based on the embryo development rate, fragmentation degree, and the parity degree of the cleavage sphere before transfer. The cleavage stage embryos categorized as grade I or II, with consistent cytoplasm and morphology, were deemed of excellent quality (19). The blastocysts were evaluated utilizing the Gardner grading system (20). Embryos with a 4BB grade or above were deemed of excellent quality.

### EMT assessment

EMT was measured using transvaginal ultrasound on the day of ET (21).

### Outcome index

EP, defined by at least one sac positioned outside the uterus, served as the main outcome measure. Additionally, the simultaneous presence of intrauterine and EP sacs was classified as EP; the EP rate was determined by dividing the number of EP cycles by the number of cycles resulting in clinical pregnancy and multiplying the quotient by 100%.

### Statistical analysis

The data were analyzed using the statistical software programs R (The R Foundation; https://www.r-project.org; version 4.2.0) and Empower Stats (www.empowerstats.net, X&Y solutions, Inc. Boston, Massachusetts). The continuous normally distributed data are reported as mean ± standard deviation while the non-normally distributed data are reported as median with the interquartile range (25th–75th percentile); the categorical variables are expressed as counts and percentages. The continuous variables were compared across different groups using the T-test for normal distributions and the Kruskal Wallis rank sum test for non-normal distributions; the categorical variables were analyzed using the chi-square test.

To assess the existence of a non-linear relationship between EMT and EP rate, smooth curve fitting and piece-wise linear regression were utilized. Univariate analysis was used to examine the factors that influence EP rate, while multiple logistic regression analysis was used to investigate the impact of EMT and other variables on EP rate while controlling for confounding factors. A nomographic prediction model was used to predict EP incidence. The predictive efficacy of the model was assessed using the area under the receiver operating characteristic (ROC) curve (AUC), employing the bootstrap sampling method for internal validation. *P* < 0.05 indicated the statistical significance threshold.

## Results

**Table 1** summarizes the essential features, cycle attributes, and outcome index of patients, revealing significant differences between the two groups in body mass index (BMI), EMT on the day of ET, tubal factors infertility, previous EP number, and ET stage (*P* < 0.05), with insignificant differences in the remaining indicators (*P* > 0.05).

**Table 1:** Essential features, cycle attributes, and outcome index of patients.

group	IUP	EP	Standardize diff.	*P*-value
N	5496	110		
Age	31.67 ± 4.17	32.11 ± 3.49	0.11 (-0.07, 0.30)	0.273
BMI	22.43 ± 3.03	23.07 ± 3.02	0.21 (0.01, 0.41)	0.037
AMH	3.24 (1.64-5.89)	3.37 (1.44-5.26)	0.08 (-0.12, 0.29)	0.556
AFC	15.00 (9.00-22.00)	15.00 (10.50-22.00)	0.02 (-0.18, 0.22)	0.773
Basal serum FSH	6.50 (5.40-8.00)	6.60 (5.70-8.07)	0.06 (-0.15, 0.27)	0.331
Basal serum E2	35.10 (24.30-48.60)	35.35 (27.60-50.48)	0.11 (-0.10, 0.32)	0.455
Duration of infertility	3.00 (2.00-5.00)	3.00 (1.38-5.00)	0.03 (-0.17, 0.23)	0.651
EMT on the day of ET	10.17 ± 1.84	9.64 ± 1.80	0.29 (0.10, 0.48)	0.003
Infertility type			0.20 (0.00, 0.40)	0.046
Primary	2744 (55.02%)	45 (45.00%)		
Secondary	2243 (44.98%)	55 (55.00%)		
Tubal factor infertility	2909 (58.50%)	89 (89.00%)	0.74 (0.54, 0.94)	<0.001
Male factor infertility	1361 (27.37%)	21 (21.00%)	0.15 (-0.05, 0.35)	0.157
Endometriosis	161 (3.24%)	1 (1.00%)	0.16 (-0.04, 0.35)	0.208
Diminished ovarian reserve	549 (11.04%)	14 (14.00%)	0.09 (-0.11, 0.29)	0.351
PCOS	762 (15.32%)	14 (14.00%)	0.04 (-0.16, 0.24)	0.716
unexplained infertility	82 (1.65%)	1 (1.00%)	0.06 (-0.14, 0.25)	0.613
Protocol			0.14 (-0.04, 0.33)	0.509
GnRHa+HRT	2542 (46.25%)	44 (40.00%)		
HRT	1297 (23.60%)	27 (24.55%)		
NC	1049 (19.09%)	23 (20.91%)		
Ovulation induction	608 (11.06%)	16 (14.55%)		
Stage of embryo transferred			0.25 (0.05, 0.44)	0.015
Cleavage	2720 (52.41%)	67 (64.42%)		
Blastocyst	2470 (47.59%)	37 (35.58%)		
Number of previous EP			0.29 (0.10, 0.49)	<0.001
0	4289 (86.49%)	75 (75.00%)		
≥1	670 (13.51%)	25 (25.00%)		
Number of embryos transferred			0.14 (-0.05, 0.34)	0.179
1	682 (13.14%)	9 (8.65%)		
2	4510 (86.86%)	95 (91.35%)		
Number of high-quality embryos transferred			0.02 (-0.17, 0.22)	0.819
0	1543 (29.74%)	32 (30.77%)		
≥1	3646 (70.26%)	72 (69.23%)		

BMI, body mass index; AFC, antral follicle count; FSH, follicle-stimulating hormone; AMH, anti-Müllerian hormone; E2, estradiol; PCOS, polycystic ovary syndrome.

**Figure 2:**
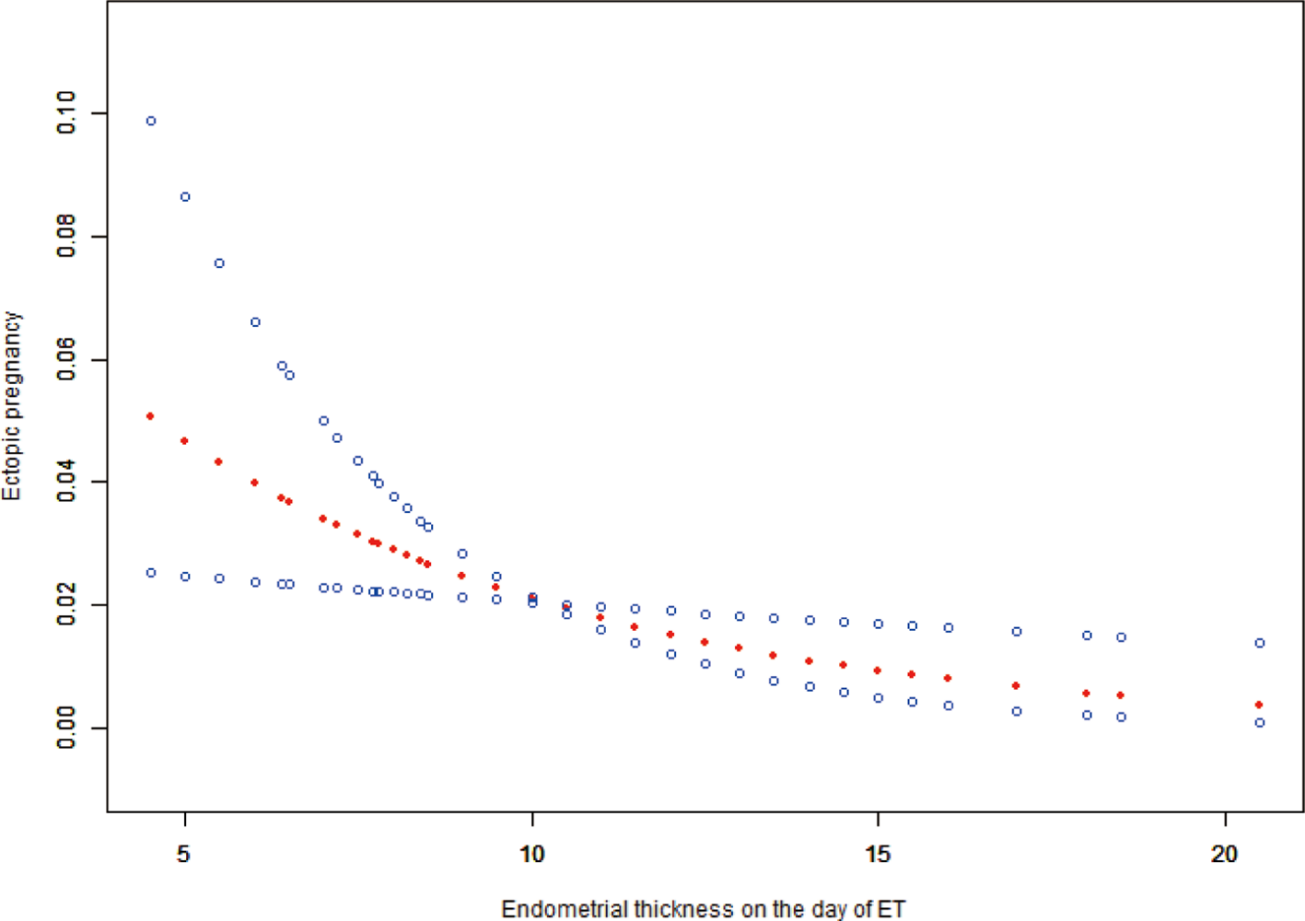
Associations between EMT on the day of ET and the EP rate. There was a non-linear association between EMT on the day of and the EP rate (*P* = 0.0106) by a generalized additive model (GAM). The smooth curve represented by the solid dotted red line effectively captures the association between the variables, while the blue curves represent the upper and lower limits of the 95% confidence interval.

After adjusting for age, anti-Müllerian hormone (AMH), BMI, basal serum follicle-stimulating hormone (FSH), infertility duration, tubal factor, type and protocol, previous EP number, ET stage and number, and high-quality ET number, the fitted curves revealed a non-linear correlation between EMT and EP rate. The EP rate decreased with an increase in EMT (**Figure 2**). **Table 2** presents the piece-wise linear regression of EMT and the EP rate. After considering the previously mentioned factors, the turning point for EMT was 9 mm; the EP rate decreased by 28% with each additional millimeter of EMT up to 9 mm (OR = 0.72; 95% CI, 0.53 to 0.99; *P* = 0.0412) while insignificantly reduced when the EMT exceeded 9 mm (OR = 0.91; 95% CI, 0.76–1.08; *P* = 0.2487).

**Table 2:** Piece-wise linear regression of EMT on the day of ET and EP.

Outcome	Turning point of EMT(mm)	Effect size (OR)	95% CI	*P-*value
ectopic pregnancy	<9	0.72	(0.53, 0.99)	0.0412
	>9	0.91	(0.76, 1.08)	0.2874

**Table 3** provides the univariate analysis results, indicating that the increased EMT on the day of ET (OR = 0.85;;95% CI, 0.76–0.94; *P* = 0.0026) and blastocyst transfer (OR = 0.61;;95% CI, 0.41–0.91; *P* = 0.0161) significantly decreased the EP risk and BMI (OR = 1.07;;95% CI, 1.00–1.13; *P* = 0.0368). Meanwhile, tubal factor infertility (OR = 3.87;;95% CI, 2.20–6.83; *P* < 0.0001), secondary infertility (OR = 1.50;;95% CI, 1.00–2.23; *P* = 0.0475), and the number of previous EP ≥ 1 (OR = 2.13;;95% CI, 1.35–3.38; *P* = 0.0012) significantly increased EP risk.

**Table 3:** Univariate analysis of factors associated with the EP rate.

Index	Ectopic pregnancy		
	OR	95%CI	*P-*value
Age	1.03	(0.98, 1.07)	0.2728
AMH	0.98	(0.93, 1.03)	0.4378
BMI	1.07	(1.00, 1.13)	0.0368
AFC	1.00	(0.98, 1.02)	0.8343
Basal serum E2	1.01	(1.00, 1.02)	0.2976
Basal serum FSH	1.01	(0.98, 1.04)	0.6238
Duration of infertility	0.99	(0.91, 1.07)	0.7574
Protocol			
GnRHa+HRT	Reference	Reference	
HRT	1.20	(0.74, 1.95)	0.4546
NC	1.27	(0.76, 2.11)	0.3630
Ovulation induction	1.52	(0.85, 2.71)	0.1561
Tubal factor infertility			<0.0001
No	Reference	Reference	
Yes	3.87	(2.20, 6.83)	
Male factor infertility			0.1588
No	Reference	Reference	
Yes	0.71	(0.43, 1.15)	
PCOS			0.7161
No	Reference	Reference	
Yes	0.90	(0.51, 1.59)	
Diminished ovarian reserve			0.3522
No	Reference	Reference	
Yes	1.31	(0.74, 2.32)	
Endometriosis			0.2348
No	Reference	Reference	
Yes	0.30	(0.04, 2.18)	
unexplained infertility			0.6159
No	Reference	Reference	
Yes	0.60	(0.08, 4.36)	
Infertility type			0.0475
Primary	Reference	Reference	
Secondary	1.50	(1.00, 2.23)	
Number of previous EP			0.0012
0	Reference	Reference	
≥1	2.13	(1.35, 3.38)	
EMT on the day of ET	0.85	(0.76, 0.94)	0.0026
Stage of embryo transferred			0.0161
Cleavage	Reference	Reference	
Blastocyst	0.61	(0.41, 0.91)	
Number of high-quality embryos transferred			0.8195
0	Reference	Reference	
≥1	0.95	(0.63, 1.45)	
Number of embryos transferred			0.1830
1	Reference	Reference	
2	1.60	(0.80, 3.18)	

The multiple logistic regression analysis included the variables with a significance level of *P* < 0.1 in the univariate analysis and all variables considered clinically relevant in potentially affecting EP incidence (**Table 4**). After adjusting for confounding factors, EP incidence significantly increased with the number of previous EP ≥ 1 (OR = 2.29; 95% CI, 1.26–4.16; *P* = 0.0064) and tubal factor infertility (OR = 3.86; 95% CI, 2.06–7.24; *P* < 0.0001). Conversely, EP risk was significantly reduced by the increase in EMT (OR = 0.84; 95% CI, 0.74–0.96; *P* = 0.0078) and blastocyst transferred (OR = 0.45; 95% CI, 0.27–0.76; *P* = 0.0027).

**Table 4:** Multivariate logistic regression analysis between EMT on the day of ET and EP.

Exposure	Model I			Model II		
	OR	(95%CI)	P value	OR	(95%CI)	*P-*value
Number of previous EP						
0	Reference	Reference		Reference	Reference	
≥1	2.21	(1.34, 3.62)	0.0018	2.29	(1.26, 4.16)	0.0064
Tubal factor infertility						
No	Reference	Reference		Reference	Reference	
Yes	4.16	(2.24, 7.71)	<0.0001	3.86	(2.06, 7.24)	<0.0001
EMT on the day of ET	0.83	(0.74, 0.94)	0.0035	0.84	(0.74, 0.96)	0.0078
Stage of embryo transferred						
Cleavage	Reference	Reference		Reference	Reference	
Blastocyst	0.47	(0.28, 0.77)	0.0026	0.45	(0.27, 0.76)	0.0027

Model I: adjusted for age, AMH, basal serum FSH, and BMI.

Model II: adjusted for all covariables in model I plus infertility duration, type, protocol, number of high-quality embryos transferred, and number of embryos transferred.

**Figure 3:**
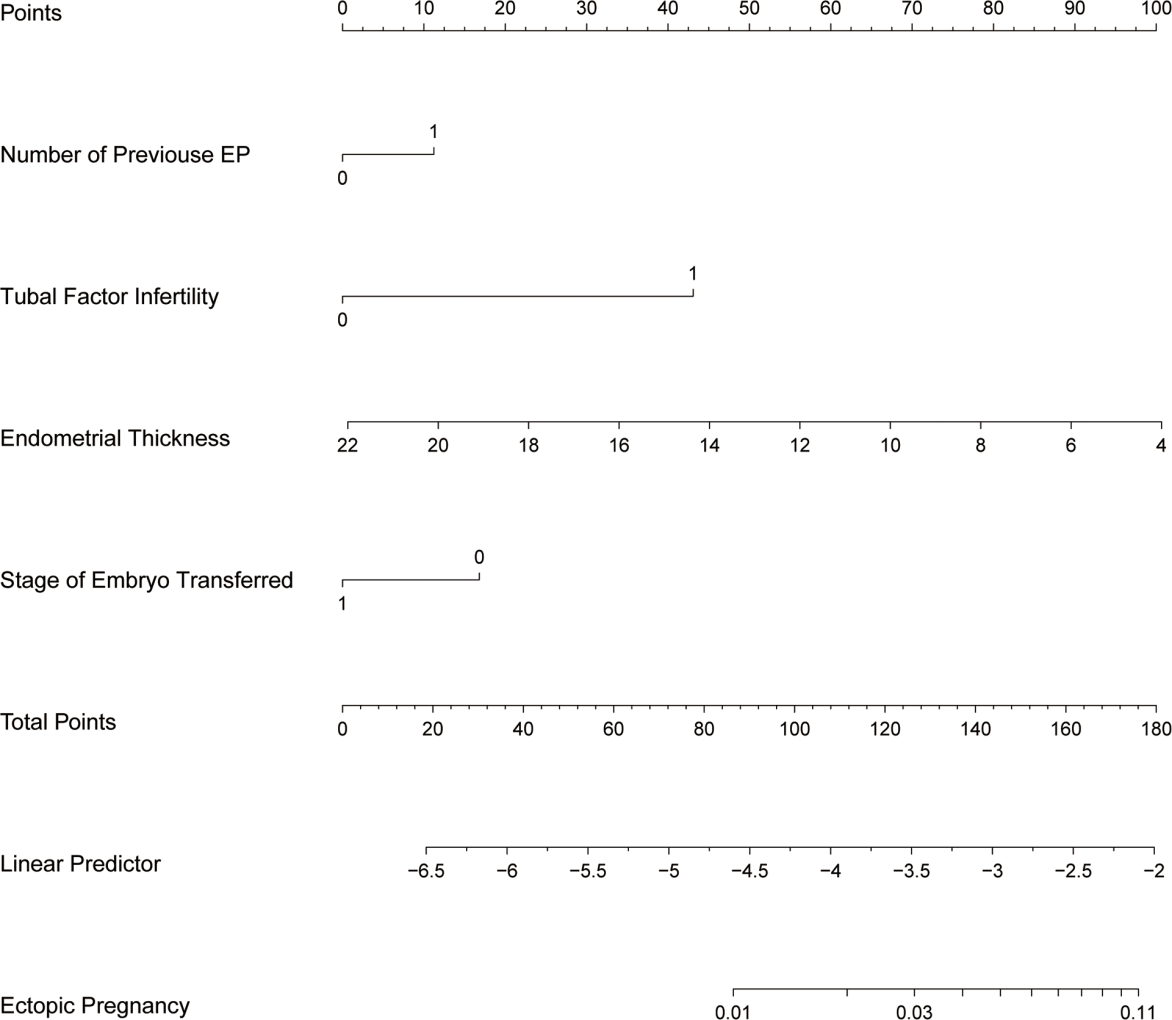
Nomogram of EP prediction model. While utilizing a nomogram, it is imperative to determine the value of each variable in a patient by referencing the corresponding point on the shaft. The variable score can be derived by extending a vertical line to intersect the score axis. Subsequently, the total score’s position can be determined by considering the scores obtained for all variables on the total score shaft.

**Figure 4:**
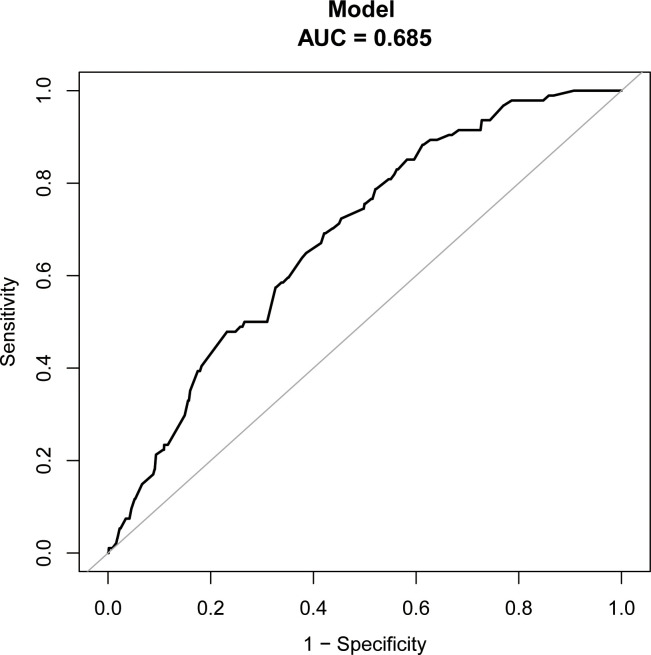
ROC curves of assessing the accuracy of EP nomogram.

**Figure 5:**
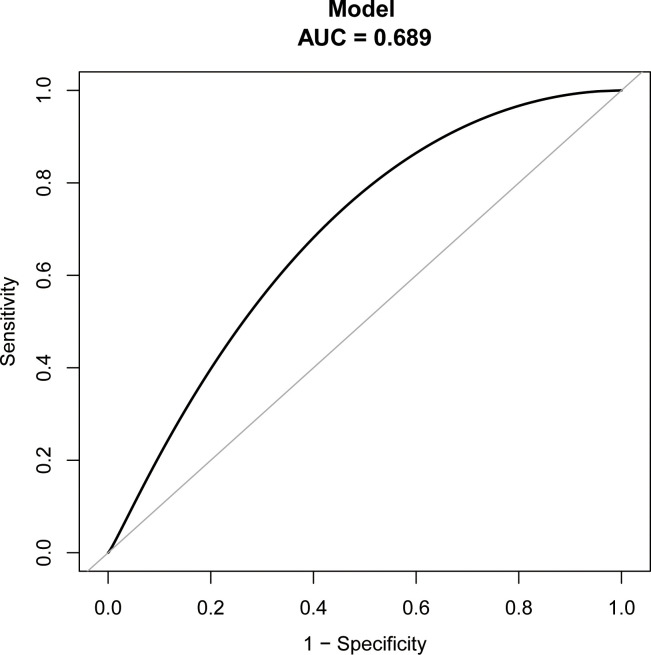
ROC curves of bootstrap resampling validation of assessing the accuracy of EP nomogram (times = 500).

The study identified the EP risk predictors, including tubal factor infertility, EMT on ET day, the ET stage, and previous EP number. These variables were selected based on the multivariate logistic regression analysis and then employed as independent variables in a nomogram prediction model (**Figure 3**). **Figure 4** illustrates the assessment of the performance of the model by calculating AUC; Logit equation [P = –3.12258–0.15524 × (EMT)–0.46983 × (blastocyst) + 0.31367 × (number of previous EP 1) + 1.20399 × (tubal factor infertility = 1)] represented the EP risk prediction model, producing an AUC of 0.685, demonstrating that the model has a moderate predictive capacity. Nomination models were internally validated using self-sampling (bootstrap sampling resampling times = 500). **Figure 5** shows that the validation resulted in an AUC of 0.689, a sensitivity of 0.6915, and a specificity of 0.5790, indicating minimal AUC fluctuations, signifying a relatively stable model.

## Discussion

In this retrospective analysis of 5606 FET cycles, a thinner endometrium on the day of ET was a separate factor in increasing EP risk. Moreover, EP was significantly associated with previous EP number, tubal factor infertility, and ET stage. Therefore, we constructed a prediction model that included these factors to predict EP incidence in individual patients before ET.

Several studies were conducted on the impact of EMT on EP rate in FET cycles. Based on reviewing 17244 FET cycles, Hongfang Liu et al. (14) determined that endometrium thinning is a separate factor in increasing EP risk, which aligns with our results. However, their study revealed that a 12 mm EMT is the critical threshold for impacting the EP rate, which was 9 mm in our study based on statistical methods. Although the results from various central data sources may differ slightly, their research measured EMT in the modified natural cycle and the mild stimulation cycle on the day of HCG injection. Moreover, they measured EMT in the HRT cycle during the final ultrasound examination before P administration.

Conversely, our study measured EMT on the transplantation day. Although their research highlighted that a prior EP occurrence could significantly elevate EP incidence, it was not incorporated or accounted for as a potential influencing factor in their study. Our study discovered that EP occurrence was influenced by previous EP number, a confounding factor, and the EMT on the day of ET posed an independent risk for EP.

Furthermore, JingLiu et al. (13) and L.Rombauts et al. (16) conducted a retrospective analysis of 12677 and 8120 cycles (comprising FET and fresh ET cycles), revealing that EMT was a separate risk factor for EP. JingLiu et al. (13) suggest that opting for a single blastocyst transfer with an EMT of at least 7.6 mm on the day of ET was preferable to decrease EP probability in fresh and FET cycles. Rombauts et al. (16) suggested that an EMT measurement of 9 mm or more significantly lower the risk of EP than an EMT measurement of less than 9 mm. The EP incidence in the fresh ET cycle was significantly greater than in the FET cycle (22–27). However, JingLiu et al. (13) and L.Rombauts et al. (16) put fresh ET and FET cycles together for statistical analysis, which may cause bias. Accordingly, we only included FET cycles to reduce the influence of confounding factors; our analysis considered and controlled for confounders, such as the endometrial preparation program and other clinical factors that could impact the EP rate. Therefore, two adjustment models were constructed to consider these factors, ensuring the stability and reliability of the results.

The reason for the increase in the incidence of ectopic pregnancy caused by endometrial thinning is not clear, and the mechanism may be complex. Some studies suggest that the thinning of the endometrium causes the implantation embryo to be closer to the uterine basilar artery, thus exposing the embryo to hyperoxia. Higher oxygen concentration will inhibit the growth of embryos (28). Lower oxygen concentration in the fallopian tube makes it easier for embryos to be implanted in the fallopian tube. This may explain that the cycle with thin endometrium has a higher risk of EP (14). Other studies have shown that endometrial thickening is positively correlated with the increased risk of placenta previa (29). It can be inferred that the thicker endometrium may be a sign of increased peristaltic waves from the floor of the uterus to the cervix (16). This may to some extent explain the reduced risk of ectopic pregnancy when the endometrium is thicker.

The advantage of our study is that combined with curve fitting and piece-wise linear regression analysis results, 9 mm was the cut-off point of EMT on the day of ET, affecting the EP rate. When the EMT on the day of ET was below 9 mm, the EP rate significantly decreased with an increase in the EMT, while it was insignificantly decreased when the EMT on the day of ET was greater than 9 mm. Therefore, the EMT should be more than 9 mm before ET to mitigate EP occurrence. Furthermore, univariate and multivariate logistic regression analyses evaluated the factors predicting EP, showing that besides the EMT on ET day, factors such as previous EP number, tubal factor infertility, and the ET stage acted as EP predictive indicators. A prognostic model was developed to estimate the rate for individual patients before ET to assess EP incidence, providing a basis for developing clinical transplantation plans.

However, our research has certain constraints. Due to the retrospective nature of this study, it was impossible to collect and account for all potential variables that may influence the results. Nevertheless, the multivariate logistic regression analysis outcomes and various adjustment models strengthen the dependability and credibility of our results. Furthermore, every ultrasound specialist at our center possesses extensive experience and conducts ultrasound scans following standard operating procedures. Although the same ultrasound physician evaluated the endometrium of each patient, potential discrepancies in the different assessments of each observer could lead to bias in our findings. We anticipate that any potential measurement discrepancy will be uniformly distributed among all participants and will not affect our main results. Finally, this study has a limited sample size, and we have no way to get data from other centers for external verification of the prediction model at present, so more samples from multicenter will need to be gathered for further research.

Based on our findings, undergoing EMT on the day of ET poses a separate risk EP in the FET cycle. The EMT should exceed 9 mm before ET to mitigate the EP incidence. Furthermore, previous EPs and tubal factor infertility were additional factors that independently increased EP risk, and implementing blastocyst transfer significantly decreased EP probability. Utilizing a nomogram predicting system allows for evaluating EP risk before ET for individual patients, providing a foundation for devising clinical strategies for ET.

## Data availability statement

The original contributions presented in the study are included in the article/supplementary material, further inquiries can be directed to the corresponding author/s.

## Author contributions

QL: Writing – original draft, Writing – review & editing. XD: Writing – original draft, Writing – review & editing. AL: Writing – review & editing. HS: Writing – review & editing. XZ: Writing – review & editing.
